# Risk factors for prolonged urine leakage following pediatric percutaneous nephrolithotomy: A comparative study of lithotripsy techniques

**DOI:** 10.1007/s00345-025-05710-5

**Published:** 2025-05-31

**Authors:** Nebil Akdogan, Mehmet Gurkan Arikan, Ismail Onder Yilmaz, Tunahan Ates, Mutlu Deger, Nihat Satar, Ibrahim Atilla Arıdogan

**Affiliations:** 1 Faculty of Medicine, Department of Urology, Cukurova University, Adana, Turkey; 2 Department of Urology, Defne State Hospital, Hatay, Turkey

**Keywords:** Percutaneous nephrolithotomy, Pediatric lithotripsy, Urine leakage, Pneumatic lithotripter, Laser lithotripter

## Abstract

**Objective:**

This study aimed to identify the risk factors associated with prolonged urine leakage (PUL) following pediatric percutaneous nephrolithotomy (PCNL) with a specific focus on the impact of lithotripter type.

**Methods:**

Data from 847 pediatric PCNL patients treated between August 1997 and February 2024 were collected. Patients were categorized into two groups based on the urine leakage time: prolonged leakage (> 24 h) and normal leakage. Logistic regression analysis was used to identify determinants of prolonged urine leakage.

**Results:**

The study found that the use of laser lithotripters (LL) significantly increased the risk of prolonged urine leakage compared with pneumatic lithotripters (PL) (OR, 3.1; 95% CI: 1.68–5.76, *p* < 0.001). Additionally, larger access diameters (≥ 26 Fr), larger stone sizes (> 350 cc), and younger age (< 6 years) were associated with an increased risk of PUL. Patients who underwent procedures with multiple accesses were also at a higher risk of PUL. Despite this, conservative management without the use of DJ stents is effective in most cases.

**Conclusion:**

The use of PL in pediatric cases is associated with a lower risk of PUL than LL. Factors such as the access diameter, stone size, and patient age can predict the likelihood of PUL. Conservative management is recommended in most patients to avoid increased morbidity.

## Introduction

In recent years, technological advancements in minimally invasive surgery have positively influenced nephrolithiasis treatment [1]. Percutaneous Nephrolithotomy (PCNL) has become the gold standard for treating stones > 2 cm in size, with a success rate of up to 90%, shorter hospital stay, and lower complication rates [1–3].

Minor complications following PCNL include hemorrhage requiring transfusion, fever, sepsis, urine extravasation, and prolonged urine leakage (PUL) from the nephrostomy tract [2–4]. PUL occurs in 1.5–4.6% of adults and 5–10% of pediatric cases [5–9]. PUL following percutaneous PCNL is defined in the literature as urine leakage persisting for more than 24 h after nephrostomy tube removal, often requiring intervention such as double-J stent placement [10]. Additionally, Seitz et al. emphasized the lack of consensus in the classification of PCNL complications but supported the clinical significance of prolonged urine leakage exceeding 24 h as an indicator of active management [6]. It is known to cause discomfort and reduce the quality of life of both physicians and patients. With the adaptation of technological innovations to PCNL, guidelines have suggested that these procedures can be used more effectively in pediatric patients [11, 12]. PCNL offers a significant advantage over open surgery, owing to its minimally invasive nature [3]. Despite these advantages, PUL remains a troublesome complication in children and occurs more frequently than in adults [6–9].

In parallel, recent years have seen a preference for lasers over pneumatic lithotripters in miniaturized instruments [11–13]. A study on stone fragment sizes found that laser lithotripters (LL) produced fragments smaller than 3 mm, whereas pneumatic lithotripters (PL) produced fragments of 3–5 mm [14]. Although pneumatic lithotripsy results in larger fragments than laser lithotripsy, these larger fragments can be retrieved using forceps to prevent obstruction by fragment migration [14, 15]. Conversely, smaller fragments produced by the laser that cannot be retrieved with forceps may lead to PUL. Not every case of PUL requires intervention, and there are conflicting data regarding whether residual fragments cause PUL [16, 17]. To the best of our knowledge, no studies have investigated the effects of laser and pneumatic lithotripsy on PUL in pediatric patients. The primary objective of this study was to assess the impact of lithotripter type on the occurrence of PUL following pediatric PCNL. The secondary objective was to examine the relationship between patient age, stone size, renal access diameter, access site, and the risk of PUL.

## Materials and methods

### Study design and study patients

This is a cross-sectional study. Data from all pediatric PCNL patients under the age of 18 years treated between August 1997 and February 2024 were collected. Postoperative urine leakage from the nephrostomy tract, either prolonged or normal, was evaluated in two groups: prolonged leakage (group 1) and normal leakage (group 2). Urine leakage was evaluated via visual inspection of the nephrostomy site after tube removal. PUL was defined as continued urine drainage beyond 24 h, confirmed by regular dressing changes every 6 h. The demographic, clinical, and biochemical data of the patients in both groups were assessed.

The inclusion criteria included all pediatric patients who underwent PCNL with either a Holmium YAG laser or a pneumatic lithotripter. Exclusion criteria included active urinary tract infection, simultaneous ipsilateral ureteral stones, irregular coagulation disorders, severe spinal deformities, and kidney anomalies.

### Surgical procedure

Urine cultures were obtained from all patients before surgery. In cases of significant microbial growth, appropriate antibiotic treatment was administered before proceeding with surgery once the culture was clear. All procedures were performed under general anesthesia. Following anesthesia induction, the patients were placed in the lithotomy position and a 5 Fr ureteral catheter was inserted. Subsequently, patients were placed in the prone position. Access to the pelvicalyceal system was achieved under fluoroscopic guidance, and the tract was dilated to 12–30 Fr over a guidewire. Tract dilation was performed using Amplatz dilators. Lithotripsy was performed using either LL or PL via a rigid nephroscope. The lithotripsy method was selected based on the stone burden and availability of equipment. Large fragments formed by the PL were removed using forceps or a basket. A 10–14 to Fr nephrostomy catheter was placed in all patients. All the procedures were performed by experienced urologists specializing in endourology.

### Ethical issue

This study was conducted following the latest version of the Helsinki Declaration and was approved by the ethics committee of our university under approval number C.U. 135/30.

### Statistical analysis

Categorical data are presented as numbers and percentages, while continuous variables are summarized using mean, standard deviation, median, and quartiles (Q1–Q3). The chi-square test was used to compare categorical variables between the groups. The Kolmogorov-Smirnov test was used to confirm the normality of the distribution of continuous variables. The Mann-Whitney U test was used to compare continuous variables between the two groups. Logistic regression analysis was conducted to identify the significant determinants of prolonged urine leakage, incorporating variables from the univariate analysis that were significant at *p* ≤ 0.25 level. All analyses were performed using the IBM SPSS 20 statistical software program (IBM Corp., Armonk, NY, IBM Corp.), with the threshold for statistical significance set at 0.05.

## Results

A total of 847 pediatric patients who underwent PCNL met the inclusion criteria. Of these, 115 (13.6%) experienced PUL. The median age was slightly lower in the PUL group compared to the normal group [6 years (IQR: 3–11) vs. 7 years (IQR: 3–13); *p* = 0.075], though the difference was not statistically significant (Table 1).

**Table 1:** Comparison of demographic and clinical characteristics between prolonged and normal urine leakage groups in pediatric PCNL patients

	Total	Prolonged Leakage (*n* = 115)	Normal Leakage (*n* = 732)	*p*
Age (years)	6 (3–11)	7 (3–13)	7 (3–12)	0.075
Age				0.165
< 6	362 (42.7%)	56 (15.5%)	306 (84.5%)	
≥ 6	485 (57.3%)	59 (12.2%)	426 (87.8%)	
Gender				
Male	486 (57.4%)	58 (11.9%)	428 (88.1%)	0.105
Female	361 (42.6%)	57 (15.8%)	304 (84.2%)	
Weight (kg)	20 (13–35)	18 (11–30)	20 (13–37)	0.055
Height (cm)	115 (90–142)	110 (82–136)	115 (90–144)	0.152
BMI	18 (15.36–20.1)	18 (16-20.2)	18 (15.7–20.2)	0.133
Stone Size (mm³)	200 (120–400)	200 (100–425)	200 (125–350)	0.624
Stone Size (mm³)				0.136
< 350	609 (71.9%)	76 (12.5%)	533 (87.5%)	
> 350	238 (28.1%)	39 (16.4%)	199 (83.6%)	

BMI: Body Mass Index, Preop: Preoperative, Postop: Postoperative, Htc: Hematocrit

*Results are expressed as percentages and median (Q1-Q3)

There were no significant differences between groups in terms of weight [18 kg (IQR: 11–30) vs. 20 kg (IQR: 13–37); *p* = 0.055], height [110 cm (IQR: 82–136) vs. 115 cm (IQR: 90–144); *p* = 0.152], or BMI [18 (IQR: 16–20.2) vs. 18 (IQR: 15.7–20.2); *p* = 0.133]. Stone size was also comparable [200 mm³ (IQR: 100–425) vs. 200 mm³ (IQR: 125–350); *p* = 0.624].

**Fig. 1:**
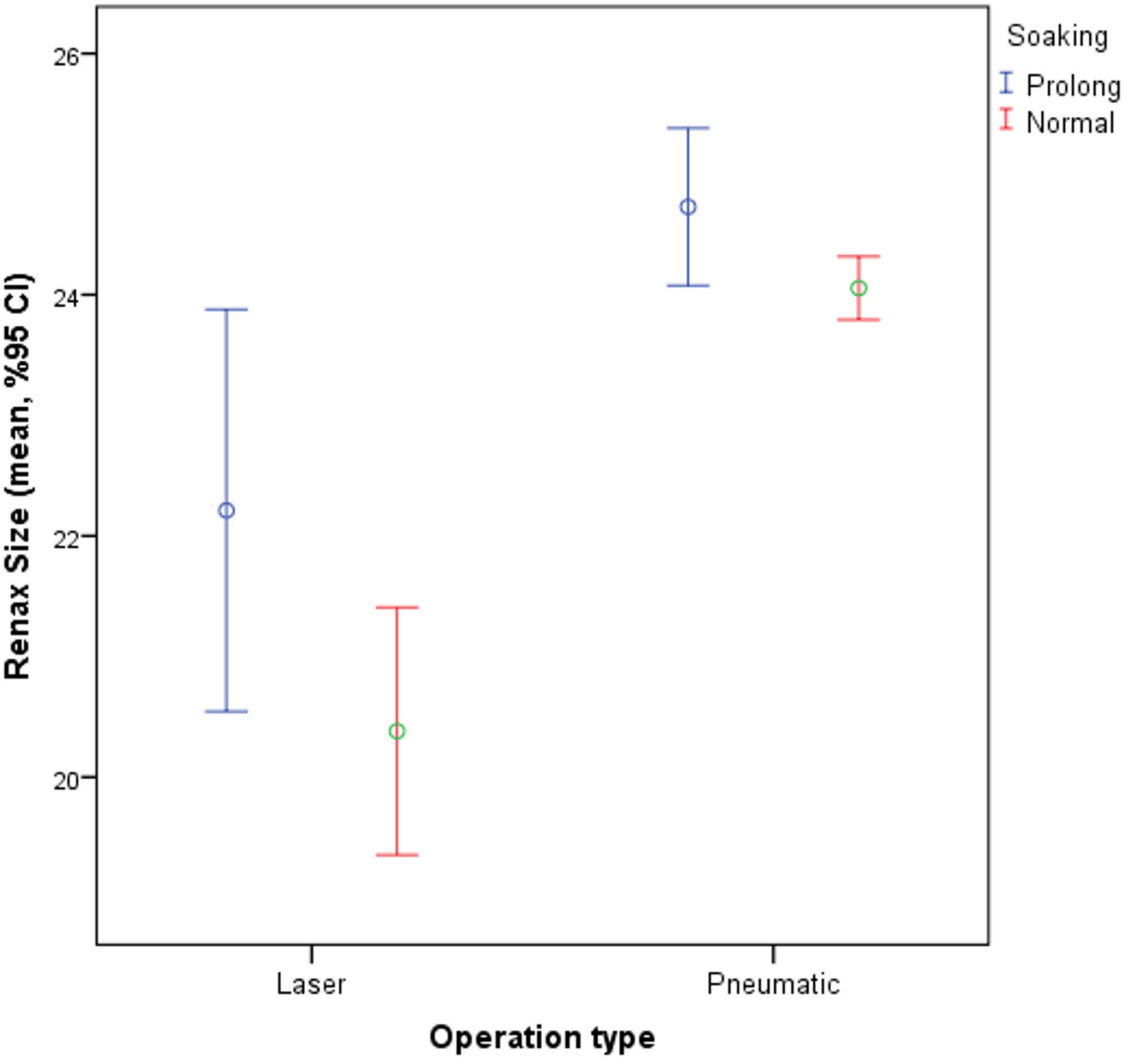
Comparison of Renal Acces Size Across Different Operation Methods and Soaking Conditions

**Fig. 2:**
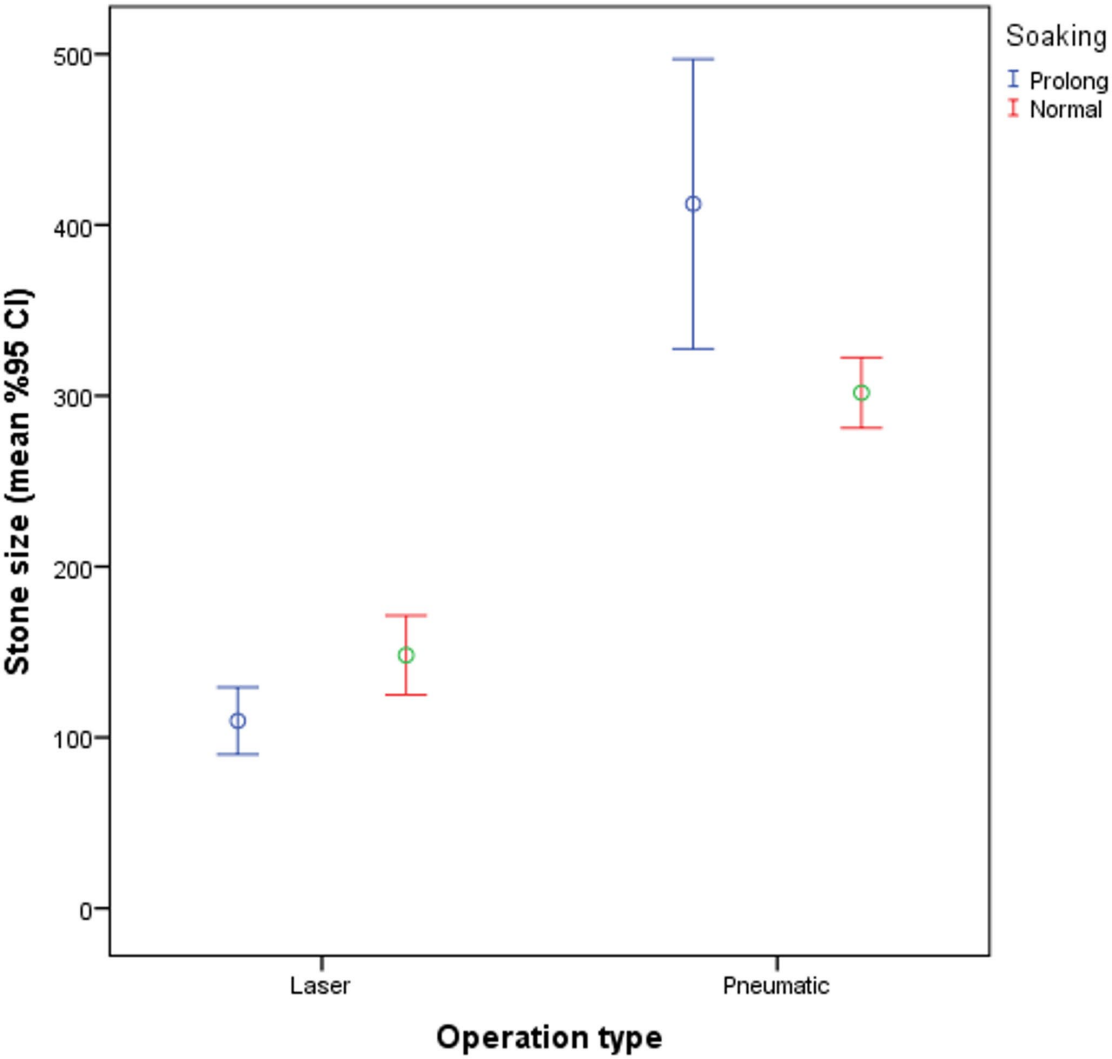
Comparison of Stone Size Across Different Operation Methods and Soaking Conditions

A significantly higher rate of PUL was observed in patients treated with laser lithotripsy compared to pneumatic lithotripsy (24.4% vs. 12.4%, *p* = 0.002). Similarly, patients who underwent procedures with multiple access tracts exhibited a higher incidence of PUL than those with single access (19.2% vs. 11.8%, *p* = 0.013). Access site (lower, middle, upper pole or pelvis) was not associated with a statistically significant difference (*p* = 0.334) (Table 2; Figs. 1 and 2).

**Table 2:** Comparison of surgical variables associated with prolonged and normal urine leakage in pediatric PCNL patients

	Total	Prolonged Leakage (*n* = 115)	Normal Leakage (*n* = 732)	*p*
**Renal Access Size (cm)**	24 (24–26)	24 (24–26)	24 (24–26)	0.179
**Renal Access Size (Fr)**				
≤ 22	97 (11.5%)	6 (6.2%)	91 (93.8%)	0.075
24	511 (60.3%)	73 (14.3%)	438 (85.7%)	
≥ 26	239 (28.2%)	36 (15.1%)	203 (84.9%)	
**Access Number**				0.013
Single	747 (88.2%)	93 (80.8%)	654 (89.3%)	
Multiple	100 (11.8%)	22 (19.2%)	78 (10.7%)	
**Access Site**				0.334
Lower Pole	490 (57.8%)	56 (11.4%)	434 (88.6%)	
Middle Pole	230 (27.2%)	31 (13.5%)	199 (86.5%)	
Upper Pole	20 (2.4%)	4 (20%)	16 (80%)	
Pelvis	7 (0.8%)	2 (28.6%)	5 (71.4%)	
**Op. Time (min)**	60 (45–95)	60 (40–100)	60 (45–95)	0.622
**Fluoroscopy Time (min)**	7 (5–12)	8 (5–12)	7 (5–12)	0.479
**Nephrostomy Size (Fr)**	14 (10–14)	14 (10–14)	14 (10–14)	0.268
**Nephrostomy Removal (days)**	2 (2–3)	2 (2–3)	2 (2–3)	0.546
**Preop Hematocrit (%)**	36 (33–39)	36 (33.4–38.5)	36 (33-38.6)	0.559
**Postop Hematocrit (%)**	33 (28.7–36.6)	34 (30-37.1)	34 (30–37)	0.349
**Preop Creatinine (mg/dL)**	0.4 (0.3–0.6)	0.4 (0.3–0.6)	0.4 (0.3–0.6)	0.382
**Postop Creatinine (mg/dL)**	0.7 (0.7–0.9)	-	0.8 ± 0.2	-
**Lithotripter Type**				0.002
Laser	82 (9.7%)	20 (24.4%)	62 (75.6%)	
Pneumatic	765 (90.3%)	95 (12.4%)	670 (87.6%)	
**Leakage Time (days)**	2 (2–3)	1 (1–1)	1 (1–1)	< 0.001
**Blood Transfusion**				0.424
No	775 (91.5%)	103 (13.3%)	672 (86.7%)	
Yes	72 (8.5%)	12 (16.7%)	60 (83.3%)	
**Residual Stones**				0.905
CIRF	87 (10.3%)	13 (14.9%)	74 (85.1%)	
CSRF	47 (5.5%)	6 (12.8%)	41 (87.2%)	
SF	713 (84.2%)	96 (13.5%)	617 (86.5%)	

Fr: French, CIRF: Clinically Insignificant Residual Fragments, CSRF: Clinically Significant Residual Fragments, SF: Stone-Free, Tx: Transfusion

*Results are expressed as percentages and median (Q1-Q3)

No significant differences were found between the two groups regarding renal access size (≤ 22 Fr vs. 24 Fr vs. ≥26 Fr; *p* = 0.075), operative time, fluoroscopy time, nephrostomy catheter size, time to catheter removal, blood transfusion requirement, or presence of residual fragments (*p* > 0.05 for all). Median urine leakage duration was significantly longer in the PUL group [2 days (IQR: 2–3)] compared to the normal group [1 day (IQR: 1–1); *p* < 0.001].

Logistic regression analysis identified four independent predictors of PUL: laser lithotripsy (OR: 3.11; 95% CI: 1.68–5.76; *p* < 0.001), renal access diameter ≥ 26 Fr (OR: 4.86; 95% CI: 1.78–13.31; *p* = 0.002), stone size > 350 mm³ (OR: 1.70; 95% CI: 1.05–2.75; *p* = 0.031), and age < 6 years (OR: 1.63; 95% CI: 1.03–2.57; *p* = 0.037) (Table 3).

**Table 3:** Logistic regression analysis of factors associated with prolonged urine leakage in pediatric PCNL patients

Variable	OR	95% CI for OR		*p*
		Lower	Upper	
**Gender (Male)**	1.43	0.95	2,15	0.086
**Age group (< 6 years)**	1.63	1.03	2,57	0.037
**BMI**	0.99	0.94	1,04	0.554
**Method (Laser)**	3.11	1.68	5,76	< 0.001
**Stone size (> 350 mm³)**	1.70	1.05	2,75	0.031
**Renal Access (24 Fr)**	3.73	1.50	9,27	0.004
**Renal Access (≥ 26 Fr)**	4.86	1.78	13,31	0.002

**OR**: Odds Ratio, **CI**: Confidence Interval

Among patients with PUL, 15 (13%) required double-J stent placement due to persistent leakage (> 72 h post-nephrostomy removal), while the remaining 87% were managed conservatively with extended external drainage and close observation.

## Discussion

Ultrasonic, pneumatic, and laser energy have been used in the treatment of urinary system stones, and the main reason for the preference for laser energy is its effectiveness in lithotripsy [14]. However, the selection of a lithotripter is an important step when considering factors such as the patient’s age, stone characteristics, device size, economic conditions of the center, and surgeon’s experience. In this study, we identified for the first time in the literature that the use of a pneumatic lithotripter in pediatric PCNL cases causes less PUL than laser. As a secondary outcome, we also demonstrated that stone size, renal access diameter, renal access site, and age were other factors that increased the incidence of PUL.

In a study by Sharma et al., which included 156 adult patients, the use of PL and LL in PCNL cases was compared, and it was reported that PL was as useful and safe as LL. Although PL was noted to be superior in terms of cost-effectiveness, no comparison has been made with PUL [15]. In our study, the incidence of PUL was lower in patients using PL. We believe this is due to the collection of fragments formed by the PL through additional maneuvers and the reduction in ureteral migration. Sharma et al.‘s study indirectly supports our findings by demonstrating that such maneuvers are used more frequently with PL [5]. Although larger fragments retrieved during pneumatic lithotripsy likely reduce the risk of ureteral obstruction and subsequent leakage, our findings and previous literature suggest that the presence of residual fragments alone is not a definitive predictor of PUL [16, 17].

In a prospective randomized study conducted in 2019, three groups of PCNL cases were compared: PL, LL, and shock pulse lithotripsy. There was no difference in the incidence of PUL among the groups, and a DJ stent was placed in patients who developed PUL [5]. In a study by Lee et al., only a small percentage of patients (1.5%) had PUL lasting more than seven days [4]. While some studies in the literature recommend placing a DJ stent in every patient with PUL lasting more than 24–48 h, it has been reported that leakage spontaneously ceases after three days with conservative follow-up [1, 7, 17, 18]. In our study, 15 patients required stenting because of PUL. Our findings support the notion that persistent urine leakage after PCNL in pediatric patients can be managed conservatively. Conservative management included mobilization, restriction of oral fluid intake, and careful monitoring of the dressings. In most cases, urine leakage resolves spontaneously within 2–3 days. The placement of a double-J (DJ) stent was considered for patients with persistent leakage lasting longer than 72 h. This approach minimizes unnecessary procedures and facilitates resolution without increasing morbidity.

In a study conducted by Rajeev et al. [5], which compared holmium laser, pneumatic lithotripsy, and Shock Pulse in percutaneous nephrolithotomy, a higher number of post-ureteroscopic lithotripsy (PUL) events were reported in the LL group than in our study. This discrepancy may be attributed to several key differences. Their study involved a pediatric population, which tends to have narrower ureters, making it easier for smaller stone fragments generated by laser lithotripsy to migrate. In addition, the average stone burden was greater in this cohort. These factors are likely to increase the incidence of fragment migration. In contrast, the use of forceps and basket maneuvers during PL may have helped prevent the migration of fragments to the ureter and other calyces.

In a study investigating the efficacy of adult PCNL instruments in pediatric cases under five years of age, PL was used exclusively, with a PUL rate of 8.2%, requiring a DJ stent [8]. Although the incidence of PUL in our study was higher, 87% of our patients managed to resolve PUL through conservative treatment alone without the need for endoscopic procedures, and no urinary system infections or other complications were observed. Our much larger sample size suggests that PUL can be managed with observation rather than immediate intervention. Although this approach may extend the hospital stay by a few days, we believe that the risks and costs associated with a new surgical procedure are lower.

In studies comparing laser lithotripsy and PL, the fragment size and migration rate were significantly higher in PL than in LL. However, when evaluating the stone-free rates (SFR) in the early postoperative period and at 3 months, the rates were similar [13–15, 19]. In our study, no significant difference in SFR was observed between the two groups. Thus, although there are differences in fragment size and migration between lithotripters, the use of PL in pediatric cases achieved a similar SFR to LL without requiring additional procedures, and statistically caused less PUL.

Fragmentation time generally affects anesthesia and surgical complications. Accordingly, as the fragmentation time increased, differences between the LL and PL emerged. LL operates through photothermal energy, and as the frequency increases, so does the temperature and tissue damage. Studies have shown that the fragmentation time is considerably longer for LL [20]. Increasing the frequency to reduce the size of fragment migration to the ureter can lead to tissue damage, which may become more complicated with prolonged time in pediatric cases with a high stone burden. When the frequency is reduced, the larger fragment sizes do not differ from the PL, resulting in increased time and cost. Additionally, lithotripsy-related damage can lead to mucosal polyps and edema [14]. In the study by Garg et al., this complication was observed in the LL group, but not in the PL group [14]. In more fragile pediatric mucosa, the development of this complication around the ureteropelvic junction (UPJ) can cause PUL due to obstruction. Since most pediatric cases in our study had a stone burden greater than 2 cm, PL stands out in light of these data.

It has been reported that the secondary endpoints of our study, such as age and increased stone size, are associated with PUL. In a study by Ansari et al. involving 936 patients, an increase in age and stone size was correlated with PUL [18]. Although this study was conducted independently of lithotripters and involved adult patients, it supports our findings. In a study by Ünsal et al. on pediatric patients, no significant difference was found between the age groups in terms of PUL [7].

The relationship between the access diameter and PUL has not been evaluated in the literature, but it is an important factor. A study evaluating the efficacy of adult instruments in children under 5 years of age during PCNL reported that these instruments are safe, and no relationship was found between instrument size and PUL [8]. In contrast, our study found that as the access diameter increased, PUL also increased, suggesting that this could be considered an independent factor.

In percutaneous nephrolithotomy, it has been shown that split renal function and complication rates with multiple accesses do not differ from those with single-access surgery [21–23]. In studies evaluating PUL among these complications, no statistically significant relationship was found between PUL and single or multiple accesses. However, in our study, PUL was observed more frequently in patients who underwent multiple accesses, and we believe that this difference is attributable to the pediatric patient group. Additionally, none of the patients who developed PUL after multiple accesses required DJ stent placement. This suggests that multiple entries in pediatric patients may increase the incidence of PUL but do not result in serious consequences requiring clinical intervention.

Prolonged hospital stays, additional tests, and surgical procedures are among the factors that negatively impact cost-effectiveness. PUL is a complication that extends the hospital stay and requires additional procedures in PCNL cases. In many developed countries, PUL is managed on an outpatient basis after discharge. In a large series of adult PCNL cases, it was reported that under existing social conditions, patients may not be discharged in this manner [16, 24]. In our study, although we hospitalized patients who developed PUL for a few days, considering the large population and the absence of urinary or wound infections, we argue that these patients can be safely managed with outpatient follow-up. However, in pediatric cases, owing to considerations regarding the mental comfort of the physician, patient, and family, hospitalization may be an option for selected cases.

Although there are conflicting data in the literature regarding cost-effectiveness, PL probes and devices, which are more durable than fragile and expensive laser probes that require frequent replacement, have been highlighted, especially in developing countries [15, 25].

### Limitations and strengths

To the best of our knowledge, this is the first study to compare the effects of PL and LL on PUL in pediatric PCNL cases with PUL evaluated as the primary outcome. This study included a large cohort of 847 pediatric patients, which strengthened the reliability of the findings. However, an important limitation of this study was its inability to perform a cost-effectiveness analysis. In addition, the retrospective nature of the study limits its ability to control for all potential confounding variables and may introduce selection bias.

## Conclusion

In this study, lithotripter type was identified as the primary factor influencing PUL in pediatric PCNL cases. PUL can be managed conservatively in most patients through extended nephrostomy drainage and observation without increasing morbidity or requiring further intervention in the majority of cases. PUL can be predicted based on access diameter, access site, age less than 6 years, and stone size greater than 3.5 cm.

## Data Availability

The data supporting this study’s findings are available on request from the corresponding author. The data are not publicly available due to privacy or ethical restrictions.
